# Data for ion and seed dependent fibril assembly of a spidroin core domain

**DOI:** 10.1016/j.dib.2015.07.023

**Published:** 2015-07-29

**Authors:** Martin Humenik, Andrew M. Smith, Sina Arndt, Thomas Scheibel

**Affiliations:** a Biomaterials, Faculty of Engineering Science, Universität Bayreuth, Universitätsstraße 30, 95440 Bayreuth, Germany; b Bayreuth Center for Colloids and Interfaces (BZKG), Universität Bayreuth, Universitätsstraße 30, 95440 Bayreuth, Germany; c Research Center Bio-Macromolecules (BIOmac), Universität Bayreuth, Universitätsstraße 30, 95440 Bayreuth, Germany; d Bayreuth Center for Molecular Biosciences (BZMB), Universität Bayreuth, Universitätsstraße 30, 95440 Bayreuth, Germany; e Bayreuth Center for Material Science (BayMAT), Universität Bayreuth, Universitätsstraße 30, 95440 Bayreuth, Germany

**Keywords:** Fibril, Nucleus, Recombinant spider silk, Seed

## Abstract

This data article includes size exclusion chromatography data of soluble eADF4(C16), an engineered spider silk variant based on the core domain sequence of the natural dragline silk protein ADF4 of Araneus diadematus, in combination with light scattering; the protein is monomeric before assembly. The assembled mature fibrils were visualized by transmission electron microscopy (TEM) and atomic force microscopy (AFM). Sonicated fibrils were used as seeds to by-pass the nucleation lag phase in eADF4(C16) assembly. We also provide data on the sedimentation kinetics of spider silk in the presence of different NaCl concentrations revealing very slow protein aggregation in comparison to the fast assembly triggered by phosphate ions published previously [1]. Experiments in the Data article represent supporting material for our work published recently [1], which described the assembly mechanism of recombinant eADF4(C16) fibrils.

**Specifications Table**

**Table:** 

Subject area	Biochemistry
More specific subject area	Structural proteins, fibril assembly
Type of data	Microscopy images, chromatograms, sedimentation kinetic data
How data was acquired	TEM, AFM, UV protein absorption, fluorescence spectroscopy multi-angle light scattering
Data format	Analyzed and processed in CorelDraw
Experimental factors	Experiments were based on soluble eADF4(C16) and tetramethylrhodamine modified eADF4(C16) in aq. buffers and assembled fibrils thereof
Experimental features	Assembly of eADF4(C16) under different salt concentration
Data source location	Biomaterials, Faculty of Engineering Science, University of Bayreuth, D-95440 Bayreuth, Germany
Data accessibility	The data are supplied with this article

**Value of the data**

Published work [1] and this additional material provide insights into how to analyze the nucleated assembly mechanism of recombinant spider silk protein eADF4(C16). The assembly mechanism of the silk compares to that of many other fibril forming proteins such as human Aβ peptides [2], huntingtin peptides [3] or yeast prion Sup35-NM [4] which all of them possess cross-β fibril structures. Data in the article and in the related publication [1] provide evidences that potassium and phosphate ions specifically trigger both nucleus formation as well as fibril growth. Both potassium and phosphate are strong kosmotropic ions and they also play a crucial role in the assembly of natural spider silks [5] in the spinning duct, whereas less kosmotropic NaCl is present in the ampulla stabilizing the protein during the storage. Therefore, the provided data could trigger further studies with amyloidogenic proteins concerning the influence of kosmotropic salts on fibril formation. In contrast, NaCl shows only marginal effect on the assembly of recombinant eADF4(C16). Nevertheless, kinetic data suggest that the self-assembly of the cross-β fibrils as described here and in [1] is not related to the assembly of natural spider silk fibers. Nucleation is accompanied by a long lag phase (hours) followed by fibril elongation possessing perpendicularly oriented β-sheets [6,7]. Natural silk assembly is very fast (in the millisecond regime) and leads to β-sheet alignment along the fiber axis [5,8].

## 1 Data

**Fig. 1:**
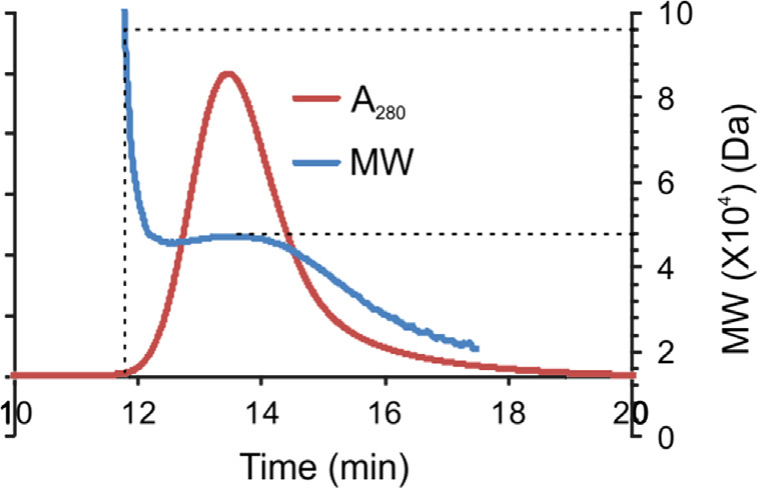
SEC-MALS analysis of soluble eADF4(C16) prepared after solubilization, dialysis and ultracentrifugation. Dashed lines show theoretical molecular weights of monomeric (48 kDa) and dimeric (96 kDa) eADF4(C16).

**Fig. 2:**
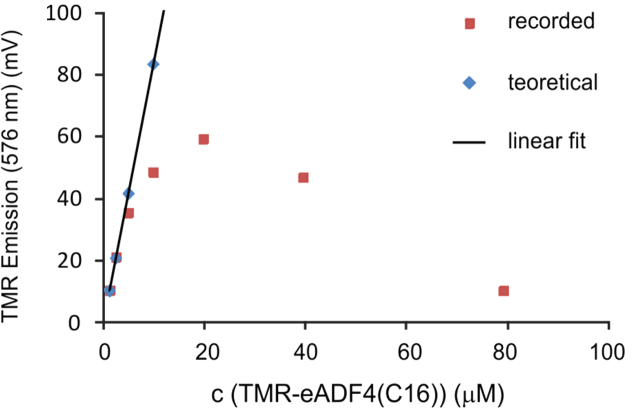
Emission intensity of tetramethylrhodamine fluorescence plotted against concentration of labeled TMR-eADF4(C16) (red squares) in comparison to the theoretical linear intensity of the dye based on the emission of labeled protein at the lowest concentration (1.2 μM) (blue diamonds) reveal a strong inner filter effect of the tetramethylrhodamine label.

**Fig. 3:**
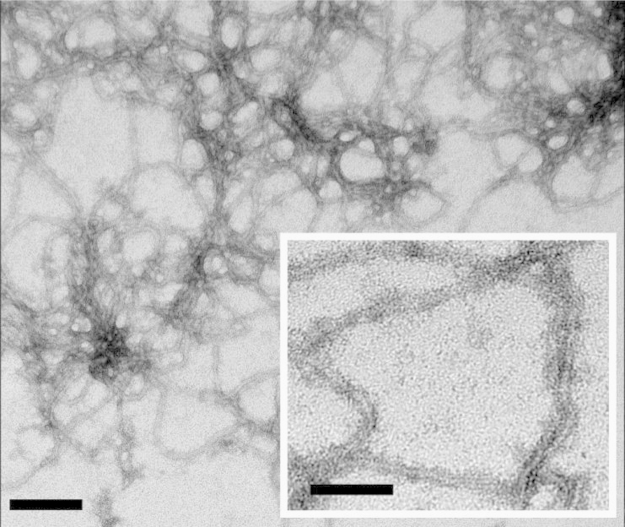
Assembly of eADF4(C16) into β-sheet rich fibrils. TEM images of air dried samples of eADF4(C16) incubated in the presence of potassium phosphate confirmed the formation of fibrillar structures with diameters below 10 nm. Fibrils were assembled at 20 μM eADF4(C16) in 100 mM K-Pi at 20 °C; Scale bars represent 100 nm for the large image and 50 nm for the insert.

**Fig. 4:**
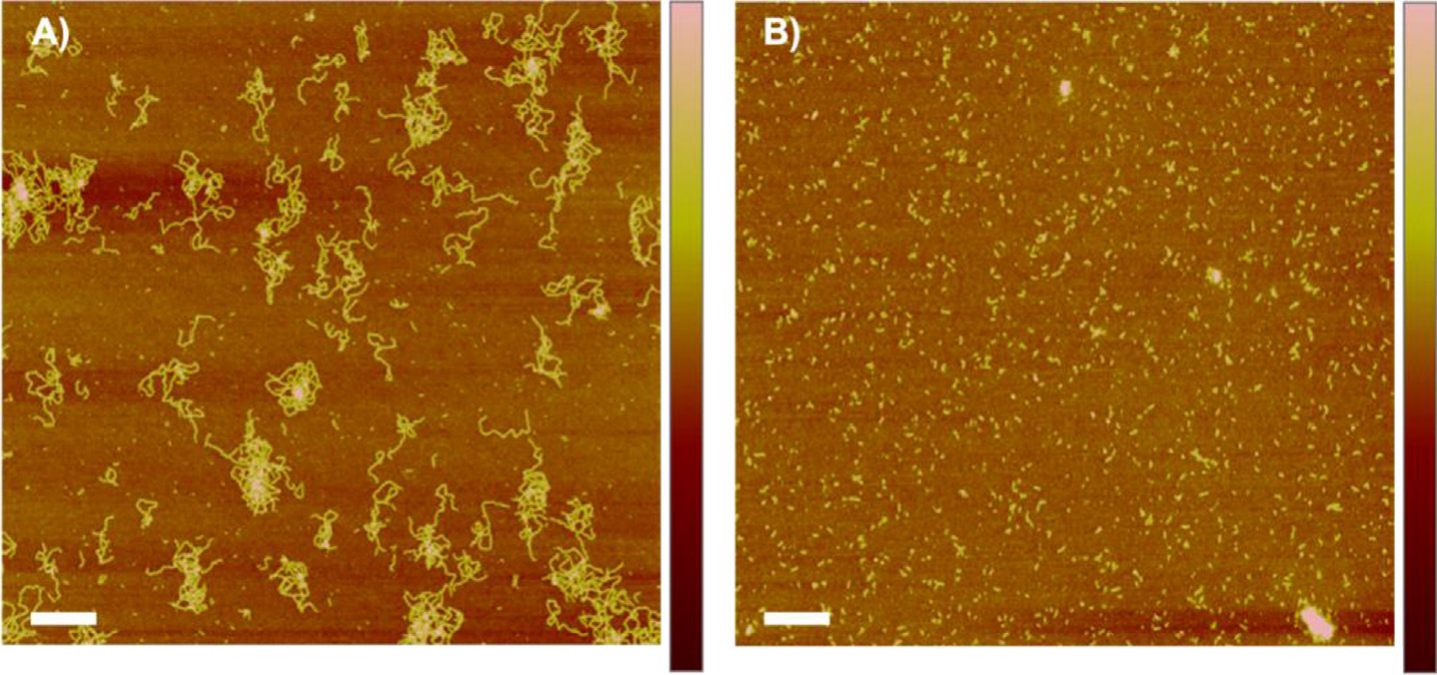
AFM scans of eADF4(C16) fibrils before (A) and after sonication (B); Scale bars 1 μm, color bars 0 nm (dark brown) – 10 nm (pale brown).

**Fig. 5:**
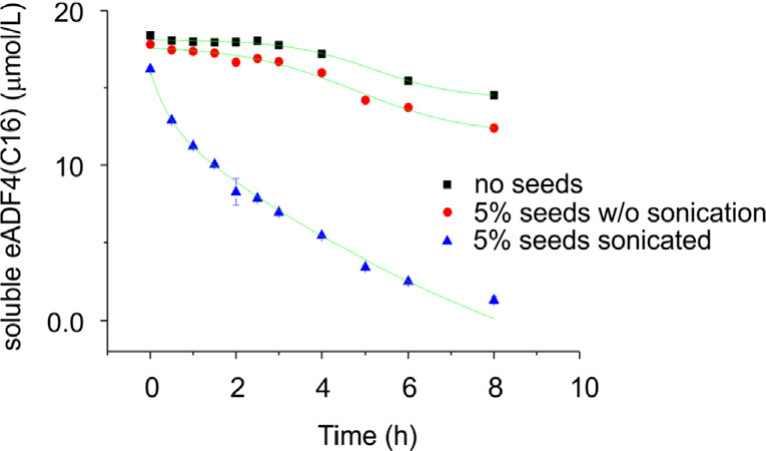
Assembly kinetics of eADF4(C16) in the presence of non-sonicated and sonicated seeds as measured by sedimentation. Soluble eADF4(C16) (20 μM) was incubated in K-Pi buffer (100 mM, pH 7.5) in the presence or absence of different seeds as indicated. At certain time points, the concentration of soluble protein was determined after ultracentrifugation using the absorption at 280 nm.

**Fig. 6:**
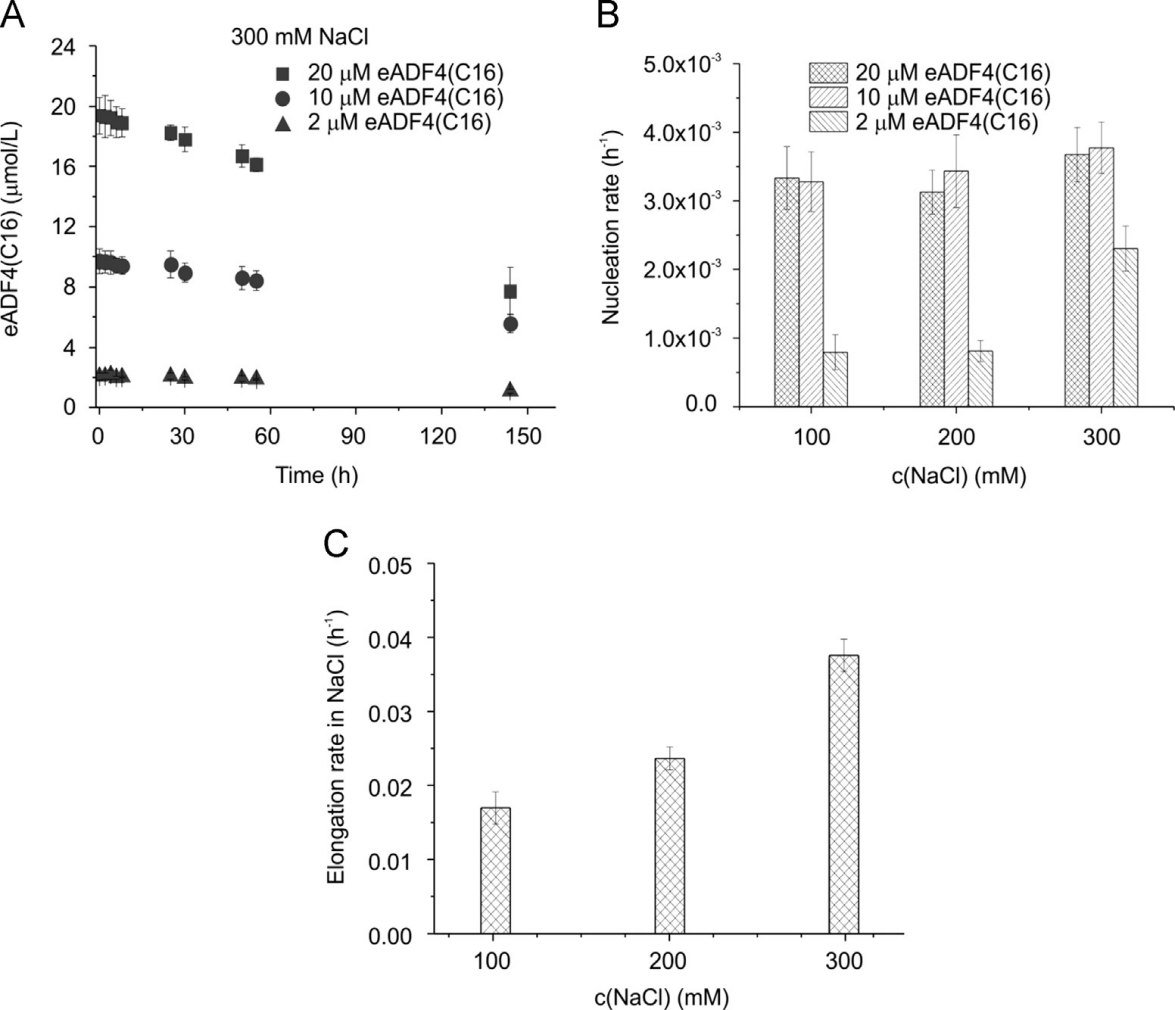
Assembly kinetics of eADF4(C16) as measured by sedimentation. (A) At certain time points, soluble protein concentrations were determined after ultracentrifugation using absorption at 280 nm. eADF4(C16) was incubated at different concentrations in the presence of different sodium chloride concentrations as exemplarily shown for 300 mM NaCl. Nucleation (B) and elongation rate constants (C) were established from assembly kinetics in (A) using the Finke-Watzky model [9]. Elongation rates are exemplarily shown for 20 μM protein at different NaCl concentrations.

In the data we included size exclusion chromatography data of soluble eADF4(C16) in a combination with light scattering revealing that the protein is monomeric before assembly (Fig. 1). A defined structural state is important before starting any kinetic study, since assemblies may significantly influence the kinetics by accelerating the nucleation (Fig. 2). The assembled mature fibrils were visualized by transmission electron microscopy (TEM) (Fig. 3) and atomic force microscopy (AFM) (Fig. 4), revealing fibrils typically 10 nm in diameter and 1 µm in lengths. Sonication led to significantly shorter fibrils, as shown by AFM, increasing the number of the active fibril ends. Sonicated fibrils can be used as seeds to by-pass nucleation in eADF4(C16) assembly (Fig. 5). We further show sedimentation kinetics of spider silk in the presence of different NaCl concentrations revealing very slow protein aggregation (Fig. 6) in comparison to the fast assembly triggered by phosphate ions [1], which indicates that ion masking events are less important for the protein-protein interation during spider silk assembly.

## 2 Material and methods

### 2.1 Multi angle light scattering (MALS) measurements

All buffers and the eADF4(C16) solution were filtered using 0.02 µm filters. The MALS measurements were carried out using an Agilent 1100 Series HPLC system connected to a multi-angle light scattering detector DAWN EOS (Wyatt, Germany). The system was additionally equipped with a Rheodyne valve and a 1 mL loop to apply the protein solution at a flow rate of 0.2 mL/min to the K5 flow cell of the MALS detector. The flow was stopped after two minutes, the inlet and the outlet tubing were sealed, and light scattering was monitored over time. Molecular masses from light scattering signals were calculated using the ASTRA 6 software (Wyatt, Germany) (Data in Fig. 1).

### 2.2 Transmission electron microscopy

A 5 µL droplet was placed on a Formvar grid and allowed to settle for 30 s before the excess solution was blotted off. Then, 2×5 µL water were added and blotted off. 5 µL uranyl acetate solution were added to the grid and left for 30 s before being blotted off. Finally, a 5 µL solution of water was added and blotted off. Samples were air dried for 16 h prior to observation on a JEOL JEM-2100 transmission electron microscope (Data in Fig. 3).

### 2.3 Atomic force microscopy

AFM scanning of dry samples was performed on a Dimension™ 3100 equipped with a NanoScope^®^ V controller (Veeco Instruments Inc., USA) operating in Tapping Mode^™^ using Si_3_N_4_ cantilevers (OMCL-AC160TS, Olympus, spring constant of 42 N/m, resonance frequency of 300 kHz, tip radius less than 7 nm). For imaging, tapping mode (ratio of setpoint amplitude to free amplitude ~0.7–0.9) was applied. AFM scans were processed using NanoScope Analysis software Version 1.40r3 (Brucker, Santa Barbara, CA) (Data in Fig. 4).
